# Supplementary figures and images for: Therapeutic candidates for keloid scars identified by qualitative review of scratch assay research for wound healing
Source: PLoS One. 2021 Jun 18;16(6):e0253669. doi: 10.1371/journal.pone.0253669 (PMC8213172; doi:10.1371/journal.pone.0253669)

Supplemental Figure 1

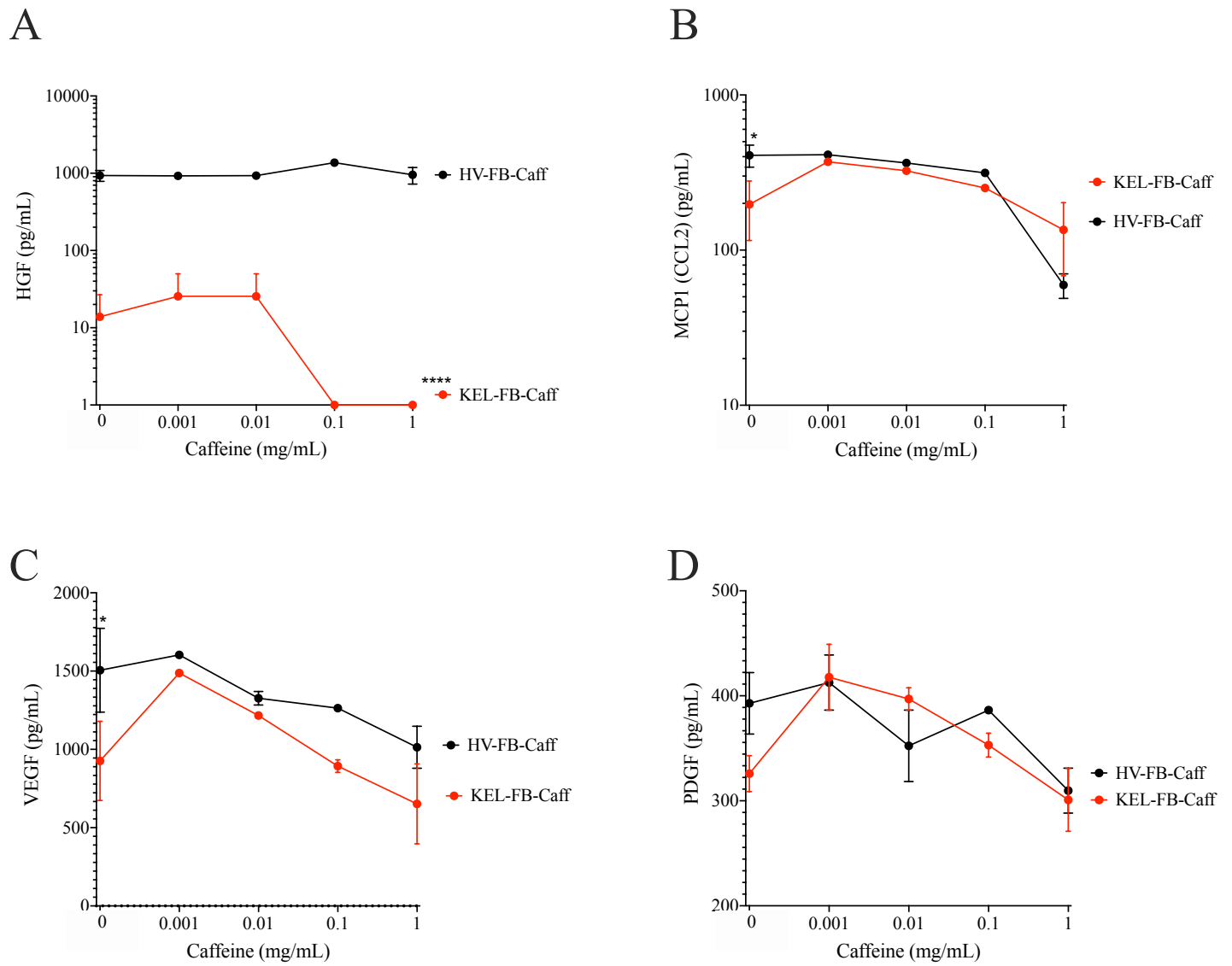

Supplement: S1 Fig — (A-D) Supernatant accumulation of HGF (A), CCL2 (B), VEGF (C) and PDGF (D) for HV-FB and KEL-FB stimulated with indicated doses of caffeine. Results are representative of three independent experiments and displayed as mean + SEM for triplicate wells. **** = p < 0.0001; for statistical comparison of area under the curve versus HV-FB under same stimulation conditions as determined by ANOVA with Sidak adjustment. (PDF) [file pone.0253669.s001.pdf]

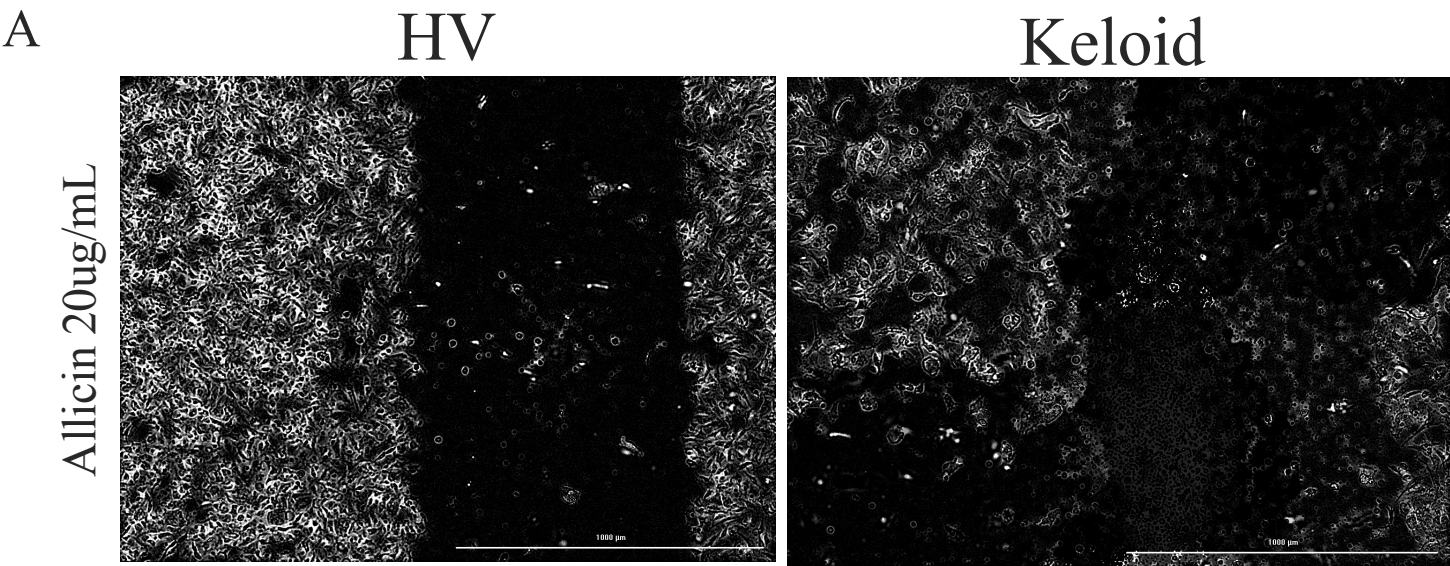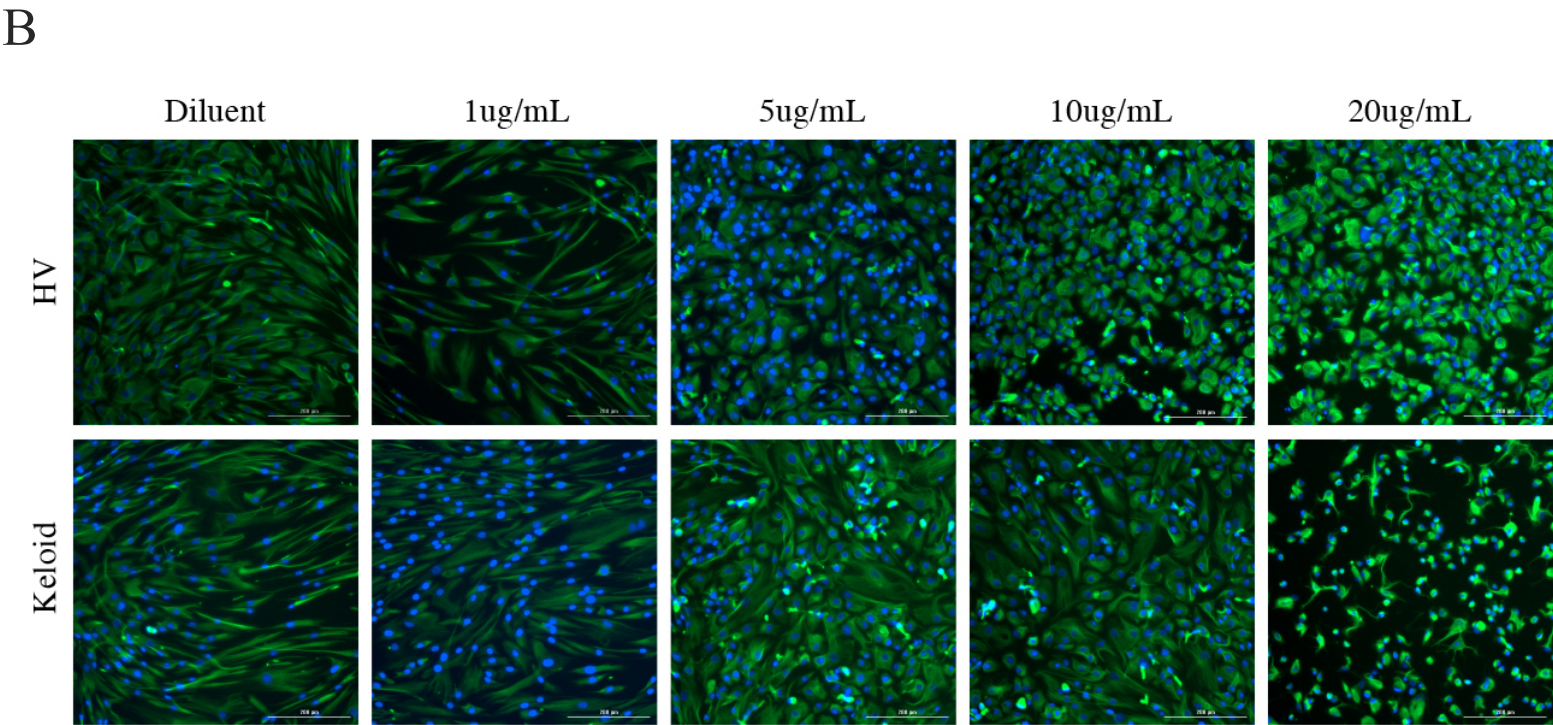

Supplement: S2 Fig — (A) Representative image for HV and KEL-FB treated with 20mg/mL allicin for 12 hours. (B) Representative images for HV and KEL-FB cells treated with indicated doses of allicin and stained for vimentin (green) and DAPI (blue). Results are representative of two independent experiments. (PDF) [file pone.0253669.s002.pdf]
